# Longitudinal ^1^H and ^129^Xe Lung MRI in Patients With Post‐COVID Residual Lung Abnormalities

**DOI:** 10.1002/jmri.70368

**Published:** 2026-05-28

**Authors:** Laura C. Saunders, Guilhem J. Collier, Laurie J. Smith, Ho‐Fung Chan, Paul J. C. Hughes, Scarlett Strickland, Lotta Gustafsson, Thomas Newman, Megan Plowright, Zoë Gabriel, Lily M. Pearce, James T. Grist, Kher Lik Ng, Arthur Harrison, Charlotte E. Bolton, Jody Bray, Helen Marshall, Graham Norquay, Alberto M. Biancardi, Jimmy E. Ball, Neil J. Stewart, Kevin M. Johnson, Andrew J. Swift, Smitha Rajaram, John Blaikley, Stefan Stanel, Paul J. Collini, Gary H. Mills, Rod Lawson, Jonathan Brooke, Amanda T. Goodwin, Iain D. Stewart, Ling‐Pei Ho, Joseph Jacob, Thomas Meersmann, Galina E. Pavlovskaya, Fergus Gleeson, Ian P. Hall, Gisli Jenkins, Jim M. Wild, A. A. Roger Thompson, the UK‐ILD consortium

**Affiliations:** ^1^ The University of Sheffield NIHR Biomedical Research Centre Sheffield UK; ^2^ 2 POLARIS, Section of Medical Imaging and Technologies, Division of Clinical, Medicine, School of Medicine and Population Health The University of Sheffield Sheffield UK; ^3^ Insigneo Institute for in Silico Medicine The University of Sheffield Sheffield UK; ^4^ Sheffield Teaching Hospitals NHS Foundation Trust Sheffield UK; ^5^ Oxford Centre for Clinical Magnetic Resonance Research University of Oxford Oxford UK; ^6^ Department of Radiology Oxford University Hospitals NHS Foundation Trust Oxford UK; ^7^ University of Oxford NIHR Biomedical Research Centre Oxford UK; ^8^ University of Nottingham NIHR Nottingham Biomedical Research Centre Nottingham UK; ^9^ University of Madison Wisconsin Madison USA; ^10^ University of Manchester Manchester UK; ^11^ National Heart & Lung Institute Imperial College London London UK; ^12^ Satsuma Lab, Hawkes Institute University College London London UK; ^13^ UCL Respiratory University College London London UK; ^14^ University of Nottingham Ningbo China

**Keywords:** 129Xe, COVID‐19, dynamic contrast enhanced, hyperpolarized gas imaging

## Abstract

**Background:**

It is unclear how lung function may recover in patients with residual lung abnormalities (RLAs) following COVID‐19 pneumonia.

**Purpose:**

To evaluate lung function trends over time in patients with RLAs following hospitalization due to COVID‐19.

**Study Type:**

Prospective, multicenter longitudinal cohort study.

**Population:**

Twenty‐four participants hospitalized due to COVID‐19 with RLAs identified on CT ≥ 3 months postdischarge (median [IQR] age 69 (15) years; 3 female) underwent at least one MRI at 6 months (*n* = 16), 1 year (*n* = 19), or 2 years (*n* = 14).

**Field Strength/Sequence:**

1.5 T. Dynamic contrast enhanced (DCE) 3D spoiled gradient echo, ^129^Xe steady state free precession (ventilation), ^129^Xe 3D spoiled gradient echo multiple *b*‐value (diffusion‐weighted), ^129^Xe 4‐echo flyback 3D radial (dissolved phase).

**Assessment:**

Pulmonary blood flow, volume, and mean transit time (MTT) were calculated from DCE MRI. The fraction of ^129^Xe signal in the red blood cells to membrane (RBC:M) was calculated from the dissolved phase ^129^Xe acquisition. Ventilation defect percentage (VDP) was calculated from the ^129^Xe ventilation acquisition. Mean diffusive length scale (Lm_D_) was calculated from the ^129^Xe diffusion‐weighted acquisition.

**Statistical Tests:**

Changes in metrics with time and associations between metrics were assessed using mixed‐effect linear regression. Correlations were tested using Spearman's correlation coefficient. Regional differences were assessed using a Friedman's test with a Bonferroni adjustment. *p* < 0.05 was considered significant.

**Results:**

Pulmonary blood flow and MTT improved significantly over time (MTT: 6 months, 15.3 (IQR, 2.0); 1 year, 15.6 (1.4); 2 years, 15.0 (5.3); pulmonary blood flow: 6 months, 75.4 (IQR, 22.0); 1 year, 83.2 (47.4); 2 years, 107.3 (51.1)). RBC:M *z*‐score was low at all three visits (6 months, −2.85 (0.98); 1 year, −2.44 (1.34); 2 years, −2.60 (1.39)), with no improvement with time (*p* = 0.993). VDP and Lm_D_ did not significantly change with time (VDP: *p* = 0.100; Lm_D_: *p* = 0.166).

**Data Conclusion:**

Improvements in lung perfusion were measured; however, there was no corresponding enhancement in RBC:M.

**Evidence Level:**

Level 2.

**Technical Efficacy:**

Stage 3.

## Plain Language Summary

Some patients hospitalized due to COVID‐19 have changes in their lungs which can last for months or years after hospitalization. This study aimed to find out whether lung function in those patients changed over time. The patients underwent a variety of MRI techniques, including a method which measures how well xenon gas can move into the blood in the lungs (gas transfer) and a method which measures blood flow in the lungs. Patients' blood flow in the lungs improved over time, but they still had abnormalities in gas transfer 2 years after hospitalization.

## 1 Introduction

Patients hospitalized due to COVID‐19 experience a persisting high symptom burden postdischarge [1]. At 5 months postdischarge, patients exhibit a greater prevalence of multiorgan abnormalities, including a higher incidence of lung MRI abnormalities compared with control groups with comorbidities [2]. Follow‐up studies in COVID‐19 survivors have reported persistent ventilation, perfusion, and transfer capacity of the lung for carbon monoxide (TL_CO_) abnormalities up to 1 year after discharge [3, 4, 5].

It is estimated that up to 11% of surviving hospitalized patients during the early COVID‐19 pandemic had residual lung abnormalities (RLAs) which may represent either early interstitial lung disease or residual pneumonitis [6], with CT and TL_CO_ abnormalities persisting at 1‐ and 2‐year follow‐up [7, 8]. Early interstitial lung disease can progress rather than resolve. While patients with progressive lung disease are likely to need more intensive follow‐up care and may benefit from antifibrotic therapies, patients whose RLAs resolve over time may require less intensive monitoring and care. It is, therefore, important to characterize functional lung impairment in patients with RLAs following COVID‐19 and whether such impairments resolve or progress over time.

^1^H and ^129^Xe lung MRI are used clinically and in research to evaluate lung function [9]. ^1^H dynamic contrast enhanced imaging enables measurement of pulmonary blood volume, pulmonary blood flow, and mean transit time, while^129^Xe lung MRI can visualize and quantify lung ventilation. Diffusion‐weighted imaging of ^129^Xe enables the measurement of the mean diffusive length scale (Lm_D_), a measure of lung microstructure [10]. ^129^Xe gas is soluble in the pulmonary membrane/tissue (membrane, M) and blood (RBCs), and the fraction of ^129^Xe MR signal measured in the airspace (gas), M, and RBCs assesses gas transfer between the three compartments [11]. Together, these methods allow a comprehensive evaluation of pulmonary function.

Patients who have had COVID‐19 have been found to have both xenon gas transfer and ventilation abnormalities [12, 13, 14], with more severe abnormalities found in hospitalized patients [15]. Although ventilation defects have been found to improve over time in patients post COVID‐19 [16], xenon gas transfer has not [17]. It is currently unclear how lung function abnormalities may progress in patients with RLAs following COVID‐19.

The aim of this work was to evaluate lung function trends over time in patients with RLAs following hospitalization due to COVID‐19, using imaging and PFT markers.

## 2 Materials and Methods

### 2.1 Study Design and Participant Recruitment

This study was approved by London‐Hampstead Research Ethics Committee (9/LO/1115), North West—Preston Research Ethics Committee (20/NW/0235) and the Tyne & Wear South Research Ethics Committee (19/NE/0330). All patients provided written informed consent.

Patients who were hospitalized due to COVID‐19 and had RLAs on their clinical chest CT or research lung MRI at ≥ 3 months after discharge were prospectively recruited from Sheffield, Manchester, Nottingham, and Oxford NHS hospitals between May 2020 and Nov 2022 as part of the XMAS study [18, p. 59]. Participants underwent MRI at either Sheffield, Nottingham or Oxford hospitals. The following inclusion criteria were applied:

- A positive SARS‐CoV‐2 result from a nasal/pharyngeal or respiratory sample
- Hospitalization with a diagnosis of pneumonia (chest x‐ray or CT scan consistent with COVID‐19 infection).
- Evidence of RLAs on CT imaging at least 3 months after hospital admission, identified by the patient's clinical radiology report generated as part of clinical care, which was confirmed later during CT scoring.
- Participant chest could fit within the ^129^Xe chest coil.

Standard MRI exclusion criteria were applied to all subjects. In addition, patients were excluded if they were unable to tolerate a test inhalation of ^129^Xe gas according to the supervising clinicians' judgment.

Participants had both ^1^H and ^129^Xe MRI examinations together at 6 months, 1 year, and 2 years after hospitalization where possible. Participants could be recruited at any of the 6‐month, 1‐year, or 2‐year time points.

### 2.2 MRI Acquisition and Analysis

Full MRI sequence details can be found in Table S1.

MRI examinations were undertaken on either a 1.5 T Signa HDx or Optima 450w (GE HealthCare, Milwaukee, WI, USA) scanner. MRI sequences were standardized across scanners. ^129^Xe doses were hyperpolarized to ~30% [19] and ^129^Xe imaging was acquired using a flexible quadrature transmit/receive vest coils (Clinical MR Solutions, Brookfield, Wisconsin, USA). For all ^129^Xe imaging sequences, ^129^Xe was administered using a bag of up to 1 L, with gas volume titrated according to subject height if subjects were < 160 cm, with inhalation starting from functional residual capacity [20].

The study followed standard MRI safety guidelines and excluded patients who were unable to tolerate a test inhalation of ^129^Xe gas according to the supervising clinicians' assessments.

^129^Xe lung ventilation (3D steady‐state free precession) MRI [21], ^129^Xe diffusion‐weighted MRI (3D spoiled gradient echo multiple b‐value sequence) [10], ^129^Xe dissolved‐phase xenon MRI (3D spectroscopic imaging of the gas and dissolved phase resonances) were acquired using the same sequence [11] and postprocessing pipeline for all sites. A structural ^1^H MR image was acquired after inhalation of a bag of air to match the lung inflation state of ^129^Xe MRI acquisitions. ^1^H dynamic contrast‐enhanced lung perfusion MRI was acquired at expiration (time‐resolved spoiled gradient echo with view sharing and parallel imaging). A half dose (0.05 mL/kg) of Gadovist (Bayer) was administered at an injection rate of 4 mL/s followed by a 20 mL saline flush at 4 mL/s. Patients were instructed to hold their breath for as long as possible and breathe shallowly thereafter.

3D radial ultra‐short echo time images [22] prospectively gated to expiration were also acquired as part of the imaging protocol to enable MR‐based visualization of lung structural abnormalities.

^129^Xe lung ventilation or ^1^H dynamic contrast enhanced lung perfusion imaging was not performed on participants scanned at Nottingham or Oxford.

Analysis of the ^129^Xe ventilation images was undertaken [23] from which ventilation defect percentage (VDP) and low ventilation percentage were calculated. The ventilation heterogeneity index of the segmented lung ventilation images was also calculated [20, 24] as a measure of the heterogeneity of the ventilated airspace.

The mean diffusive length scale (Lm_D_, a metric of mean acinar airway dimensions) was calculated for each voxel of the ^129^Xe diffusion‐weighted images using a stretched exponential model of ^129^Xe gas diffusion [25] and the standard deviation of the mean diffusive length scale (Lm_D_ SD) was also calculated as a measure of acinar airway dimension heterogeneity.

Maps of the fraction of xenon signal in the RBCs to airspace (RBC:gas), membrane to airspace (M:gas), and RBCs to membrane (RBC:M) were calculated from the 3D ^129^Xe dissolved phase images [11]. *Z*‐scores were calculated for each metric based on published data from a cohort of 62 healthy participants [26]. The transverse relaxation time (*T*_2_*) of the ^129^Xe in the membrane and RBC was calculated using a triple Lorentzian fit of the data in the frequency domain.

Perfusion time‐course images were coregistered to correct for patient motion using ANTs software [27] and for each voxel, signal was smoothed via Savitzky‐Golay filtering and voxel‐wise concentration maps were calculated using relative signal enhancement. The arterial input function was defined using a manual region of interest in the pulmonary artery. Semiquantitative maps of relative pulmonary blood volume, relative pulmonary blood flow and mean transit time were calculated [28], hereafter referred to as pulmonary blood volume and pulmonary blood flow for brevity. The median and interquartile range (IQR) of each whole‐lung map were calculated, with the IQR of each map hereafter referred to as spatial heterogeneity, that is, pulmonary blood volume spatial heterogeneity. Segmentations excluding main pulmonary vessels were applied to the maps.

All images underwent a quality‐control evaluation by a team of experienced ^129^Xe and lung perfusion MRI physicists and medical image software developers (experience range: 10–15 years), where images with low SNR, image artifacts, or significant respiratory motion were excluded from analysis.

### 2.3 CT Acquisition and Analysis

CT imaging was acquired as part of patients' routine clinical care at each institution and was retrospectively accessed for scoring. CTs were contrast‐enhanced if clinical need required it (*n* = 6). All CTs were performed at inspiration breath hold.

For CT scans acquired in Sheffield (*n* = 17), all CTs were volumetric acquisitions performed on either a TOSHIBA Aquilion PRIME (*n* = 6), Canon medical Systems Aquilion ONE (*n* = 11), at 1.0 mm slice thickness and a 512 × 512 acquisition matrix with standard reconstruction. KVP was either 100 or 120 kV and collimation was 1.00 mm. Resultant exposure was between 47 and 206 mA. For CT scans acquired in Nottingham (*n* = 3), scanner manufacturer was unavailable; all scans were acquired with either 1.0 mm (*n* = 2) or 2.0 mm (*n* = 1) slice thickness and a 512 × 512 acquisition matrix. For CT scans acquired in Oxford (*n* = 3), all CT acquisitions were performed at 1 L inspiration breath hold on a GE scanner with slice thickness 0.625 mm. Information on acquisition was unavailable for one CT scan.

Research ethics enabled sharing of clinical data but not transfer of clinical CT images to another site for scoring. CTs were therefore scored by a different individual at each site. CT scorers were a chest radiologist (SR, 14 years' experience), chest physician (AG, 11 years' experience; KLN, in‐training) depending on the site.

CTs were assessed for the following pulmonary abnormalities: total lesions, ground glass opacities, consolidation, reticulation, and fibrotic‐like changes (traction bronchiectasis, parenchymal bands, and/or honeycombing). A semiquantitative CT score was assigned based on the area involved in each of the five lung lobes: 0 (no involvement), 1 (< 5%), 2 (5%–25%), 3 (26%–49%), 4 (50%–75%), and 5 (> 75%). The total CT severity score was calculated by summing the individual lobar scores (possible range from 0 to 25) [29].

### 2.4 Pulmonary Function Testing (PTF)

Whenever possible, participants underwent same‐day PFTs. Forced expiratory volume in 1 s (FEV_1_), forced vital capacity (FVC), FEV_1_/FVC, the transfer factor for carbon monoxide (TL_CO_), and the carbon monoxide transfer coefficient (K_CO_) were measured and presented as *z*‐scores [30, 31]. If same‐day PTFs were unavailable, the most recent routine outpatient PFT within 4 months of the MRI was used. For participants with multiple visits, outpatient PFTs were matched to the MRI visit closest to their acquisition.

### 2.5 Statistical Analysis

The primary outcome of this study was whether longitudinal change could be measured from functional lung MRI metrics, which was assessed using mixed‐effect linear regression using a random intercept, with Maximum Likelihood estimation in IBM SPSS Statistics 27 (SPSS, New York, USA). Regression models were adjusted for age and sex if not already adjusted via *z*‐scores, and ventilation metrics were also adjusted for smoking status.

A secondary outcome of this study was whether associations between metrics could be established, which was assessed using repeated measures mixed‐effect linear regression using a random intercept.

Within‐subject tests for differences between upper, middle, and lower regions of RBC:M, M:gas, RBC:gas, and Lm_D_ were performed at all three visits (6 months, 1 year, 2 years) using a Friedman's test with a Bonferroni adjustment for multiple testing and denoted *p*_adj._ CT scores were tested for correlation with MRI and PFT metrics at 6 months using Spearman's correlation coefficient; MRC breathlessness score was tested for correlation with MRI and PFT metrics at 3 months, as well as CT data at 6 months, using Spearman's correlation coefficient.

Acute hospital admission duration was correlated with CT metrics and 6‐month MRI and PFT metrics.

Data are presented as median (IQR) unless otherwise specified. For data presented as *z*‐scores, data < −1.64 are considered abnormal. A sample size calculation was not performed because this was an exploratory study. *p* < 0.05 was considered statistically significant.

## 3 Results

**FIGURE 1:**
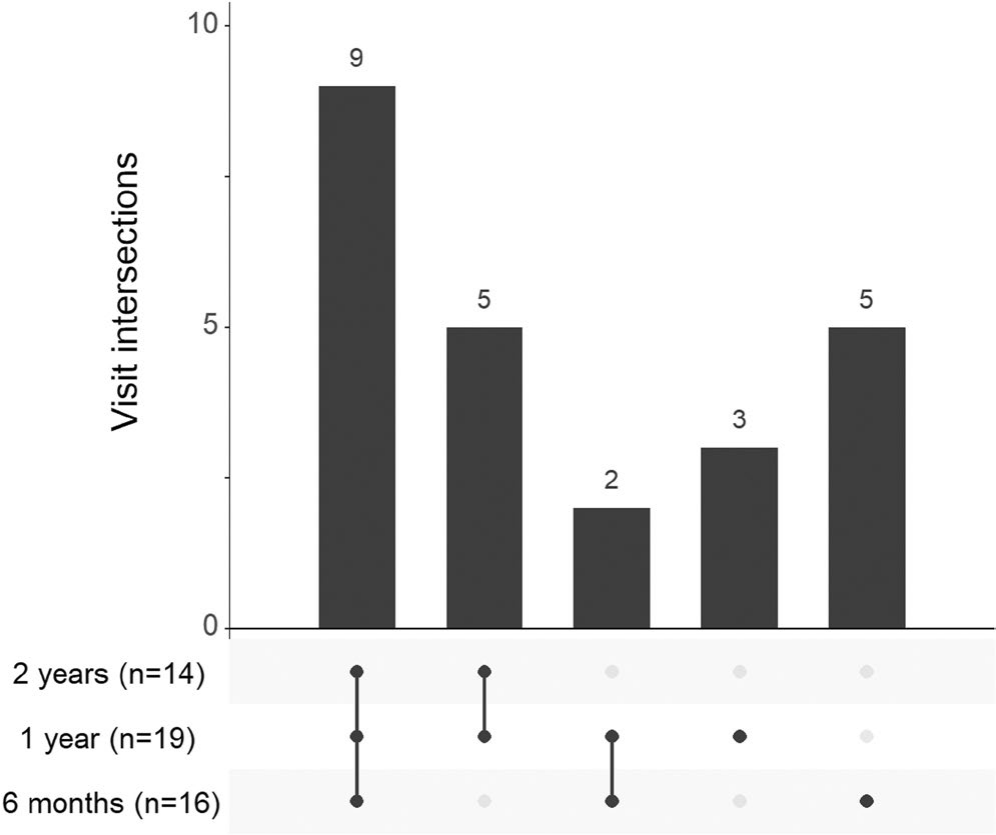
A diagram showing participant recruitment. Participants were recruited at both 6 month and 1 year visits. One patient follow‐up was excluded as it was performed on a 3 T field strength scanner.

A total of 26 participants were recruited and underwent MRI. Of the 26 participants, 1 was not included in this analysis as they were not hospitalized due to COVID‐19 and 1 participant did not have images of sufficient quality for analysis. Therefore, 24 participants were included in the final analysis. MRI visits were undertaken by 16 participants at 6 months, 19 participants at 1 year and 14 participants at 2 years, see Figure 1. Nine participants attended all three visits, two participants attended 6 month and 1 year visits only, five participants attended 1 year and 2 year visits only, five participants attended the 6 month visit only, and three participants attended visit 1 only. Full details of participants' visits and missing data are given in Table S2.

Of the 24 participants, 21 were male with a median age of 69 (15) years and BMI of 31 (6) kg/m^2^. Smoking history was documented for 22/24 participants; 10 of those participants did not smoke, and 12 participants either did smoke or had previously smoked. Of the 24 participants, 9 had severe acute disease (World Health Organization score ≥ 6) during their initial hospitalization [32]. Table 1 gives further demographic and clinical information.

**TABLE 1:** Participant demographics and clinical information.

Variables	Median (range)
Age (years)	69 (53–79)
Sex, *N* (%)	21 male, 3 female
BMI (kg/m^2^)	30.4 (20.4–38.9)
Length of stay (weeks)	4 (0–17)
Year of initial hospitalization	15
2020	9
2021	
ISARIC 4C score^a^	9 (4–18)
MRC breathlessness score (6 weeks)^b^	3 (2–5)
MRC breathlessness score (3 months)^c^	3 (1–5)
MRC breathlessness score (1 year)^b^	3 (1–5)

	Group	Number of patients
Prior respiratory history	No previous respiratory conditions	22
	Asthma	2
Comorbidities 4C score (2)	0	10
	1	5
	≥ 2	9
World Health Organization score	4 (Hospitalized, oxygen mask or nasal prongs)	11
	5 (Hospitalized, noninvasive mechanical ventilation or high‐flow nasal cannula)	4
	6 (Hospitalized, intubation and invasive mechanical ventilation)	8
	7 (Hospitalized, invasive mechanical ventilation + additional support such as pressors or extracardiac membranous oxygenation)	1
Tobacco use history	Unknown	2
	Nonsmokers	10
	Ever‐smokers	12
Treatment during admission	Antibiotics	16/24
	Dexamethasone	19/24
	Prednisolone	2/24
	Remdesivir	13/24
	Tocilizumab	3/24
	Baricitinib	1/24
	Plasma	2/24
	Treatment dose dalteparin	12/24
	Colchicine	0/24
	Aspirin	0/24

Abbreviations: BMI = body mass index; ISARIC = International Severe Acute Respiratory and Emerging Infection Consortium; MRC = Medical Research Council.

^a^ 23 patients.

^b^ 12 patients.

^c^ 19 patients.

### 3.1 Patient Symptoms

Median MRC breathlessness score at 6 weeks, 3 months, and 1 year postdischarge was 3 (range, 2–5), 3 (1–5), and 3 (1–5). Of the 14 participants with multiple MRC breathlessness scores available, 6 showed an improvement in score and 8 showed no change in score. MRC breathlessness score did not worsen in any patients with available data.

MRC breathlessness score at 3 months significantly correlated with 3 month RBC:M *z*‐score (*r* = −0.85, *n* = 13), RBC:gas *z*‐score (*r* = −0.84, *n* = 13), and TL_CO_ *z*‐score (*r* = −0.68, *n* = 10). No other significant correlations with MRI or PFT metrics were found.

MRC breathlessness score at 3 months correlated significantly with reticulation score at 1 year (*r* = 0.51, *n* = 18). No other significant correlations with CT data were found.

### 3.2 CT

CT scoring was available for 23/24 participants, median time between CT and hospital discharge of 5 (3) months. One participant had CT imaging available for confirmation of adherence to the inclusion criteria, but the data was not able to be scored. Ground glass opacities were present on CT in 21/23 participants, reticulation was present in 22/23 participants, and fibrosis was present in 10/23 participants. Additional details on CT scores are given in Figure S1.

CT scores were correlated with 6‐month MRI data. Ground glass opacity score correlated with mean transit time (*r* = −0.652) and K_CO_ *z*‐score (*r* = 0.650), see Figure S2. Reticulation score correlated positively with Lm_D_ standard deviation (SD) (*r* = 0.611), negatively with M:gas *z*‐score (*r* = −0.557), and negatively with FVC *z*‐score (*r* = −0.704). Fibrosis score correlated with TL_CO_ *z*‐score and K_CO_ *z*‐score (*r* = −0.695 and *r* = −0.608, respectively); however, only three participants with TL_CO_ and K_CO_ *z*‐score had non‐zero fibrosis scores so this should be interpreted with caution. Total CT score correlated negatively with RBC T_2_* (*r* = −0.631). No other significant correlations between CT scores and MRI or PFT metrics were found.

### 3.3 PFTs

PFT data were available for 13/16 participants at 6 months, 18/19 participants at 1 year, and 10/14 participants at 2 years. One participant could not achieve a reliable FVC measurement at their 1 year visit due to cough, and their FVC measurement was excluded. Data at visit are summarized in Table 2.

**TABLE 2:** Summary of MRI and PFT parameters at 3 timepoints.

MRI or PFT metric	6 months		1 year		2 years	
	*N*	Median (IQR)	*N*	Median (IQR)	*N*	Median (IQR)
Time since discharge (weeks)	16	23 (7.5)	19	52 (10)	14	101 (10)
Time between MRI and PFT (days)	13	16 (31)	18	0 (17)	10	0 (0)
FEV_1_ *z*‐score	13	−1.31 (0.80)	18	−0.84 (1.76)	10	−0.87 (0.61)
FVC *z*‐score	13	−1.60 (0.82)	17	−1.04 (1.57)	10	−1.18 (0.88)
FEV_1_/FVC *z*‐score	13	0.52 (0.84)	17	0.30 (1.23)	10	0.56 (1.31)
TL_CO_ *z*‐score	13	−2.19 (2.24)	18	−2.49 (1.64)	10	−2.50 (1.64)
K_CO_ *z*‐score	13	−1.12 (1.56)	18	−0.96 (1.62)	10	−0.73 (1.16)
VDP (%)	13	1.4 (1.4)	12	2.0 (2.1)	12	2.2 (3.6)
Low ventilation percentage (%)	13	14.0 (2.4)	12	14.5 (3.7)	12	13.9 (4.2)
Ventilation heterogeneity index	13	9.3 (2.3)	12	9.6 (2.0)	12	9.2 (1.5)
Lm_D_ (μm)	16	294 (19)	14	295 (23)	12	295 (26)
Lm_D_ SD (μm)	16	68.8 (12.8)	14	64.5 (11.3)	12	64.6 (11.2)
RBC:M	14	0.19 (0.04)	17	0.20 (0.08)	13	0.22 (0.06)
RBC:M *z*‐score	14	−2.85 (0.98)	17	−2.44 (1.34)	13	−2.60 (1.39)
M:gas	14	0.011 (0.022)	17	0.009 (0.005)	13	0.010 (0.003)
M:gas *z*‐score	14	0.86 (1.06)	17	0.12 (2.24)	14	0.52 (1.53)
RBC:gas	14	0.0021 (0.0012)	17	0.0016 (0.0007)	13	0.0020 (0.0009)
RBC:gas *z*‐score	14	−0.96 (2.01)	17	−1.58 (1.27)	13	−0.82 (1.00)
M T_2_* (ms)	14	2.61 (0.21)	17	2.57 (0.21)	13	2.53 (0.19)
RBC T_2_* (ms)	14	2.41 (0.21)	17	2.34 (0.27)	13	2.24 (0.37)
Pulmonary blood volume (a.u.)	11	19.6 (7.2)	15	22.1 (10.7)	9	22.7 (9.5)
Pulmonary blood volume spatial heterogeneity (a.u.)	11	20.2 (8.2)	15	19.4 (10.7)	9	21.4 (10.8)
Pulmonary blood flow (a.u.)	11	75.4 (22.0)	15	83.2 (47.4)	9	107.3 (51.1)
Pulmonary blood flow spatial heterogeneity (a.u.)	11	79.1 (42.5)	15	76.0 (48.9)	9	96.0 (46.4)
Mean transit time (s)	11	15.3 (2.0)	15	15.6 (1.4)	9	15.0 (5.3)
Mean transit time spatial heterogeneity (s)	11	1.7 (0.9)	15	1.3 (0.6)	9	1.1 (0.4)

Abbreviations: FEV_1_: forced expiry volume in 1 s; FVC: forced vital capacity; IQR: interquartile range; KCO: carbon monoxide transfer coefficient; LMD: mean diffusive length scale; M: membrane; PFT: pulmonary function tests; RBC: red blood cell; SD: standard deviation; TLCO: transfer factor for carbon monoxide; VDP: ventilation defect percentage.

Spirometry showed abnormalities consistent with a restrictive pattern, with abnormal FVC *z*‐score in 5/13 participants at 6 months, 3/18 participants at 1 year, and 2/10 participants at 2 years. One participant had spirometry consistent with an obstructive lung disease pattern (abnormal FEV_1_/FVC *z*‐score) at 6 months.

**FIGURE 2:**
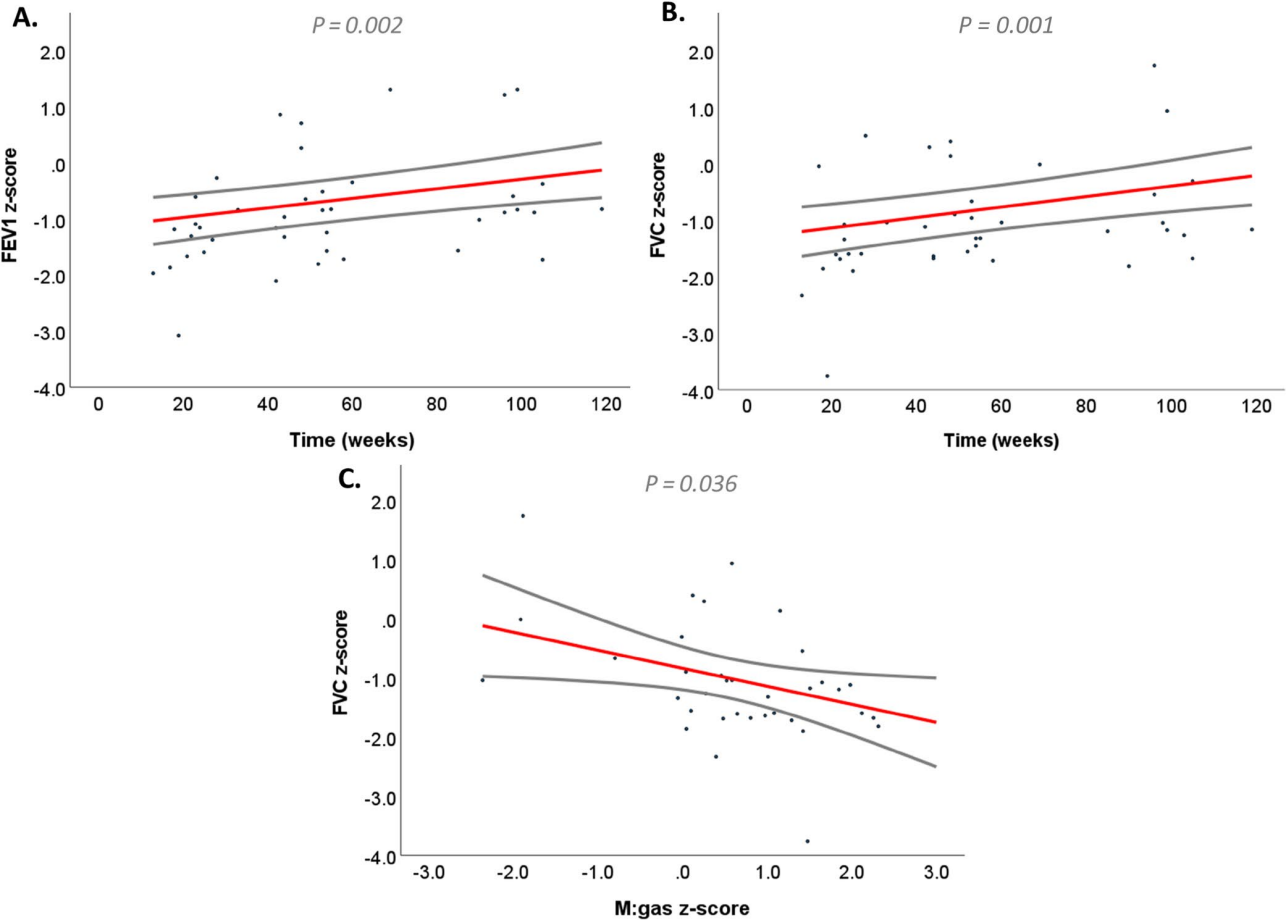
Scatter plots of spirometry lung volume (FEV_1_ and FVC *z*‐scores) plotted with time (A, B) and with M:gas *z*‐score (C), with solid lines indicating the linear trend and confidence intervals predicted by the mixed‐effect linear regression analysis.

Lung volumes significantly increased toward normal values with time (Table 3 and Figure 2), with an increase of FVC *z*‐score and FEV_1_ *z*‐score of 0.009 and 0.009 per week, respectively, which equates to an increase of 0.7 and 0.7, respectively, during the 18 month time frame of the study.

**TABLE 3:** Effect of time (measured in weeks) on MRI and PFT metrics tested using a mixed‐effect linear regression analysis with a random intercept.

Independent variable	Estimated coefficient	Lower 95% CI	Upper 95% CI	*p*
FEV_1_ *z*‐score	0.009	0.004	0.013	0.002
FVC *z*‐score	0.009	0.004	0.014	0.001
FEV_1_/FVC *z*‐score	−0.002	−0.009	0.005	0.633
TL_CO_ *z*‐score	0.005	−0.001	0.011	0.087
K_CO_ *z*‐score	−0.001	−0.005	0.003	0.550
VDP (%)	0.009	−0.002	0.020	0.100
Low ventilation percentage (%)	−0.014	−0.029	0.004	0.057
VHI	−0.004	−0.011	0.002	0.194
Lm_D_ (μm)	−0.093	−0.228	0.041	0.166
Lm_D_ SD (μm)	−0.065	−0.105	−0.025	0.003
RBC:M *z*‐score	−0.024 × 10^−2^	−0.005	0.005	0.993
RBC:M	−0.013 × 10^−3^	−0.000309	0.000282	0.926
RBC:gas *z*‐score	0.016 × 10^−1^	−0.0069	0.0102	0.696
RBC:gas	0.008 × 10^−4^	−0.000006	0.000004	0.735
M:gas *z*‐score	0.026 × 10^−1^	−0.0052	0.0105	0.490
M:gas	0.006 × 10^−3^	−0.000011	0.000022	0.490
M T_2_* (ms)	−0.008 × 10^−1^	−0.0017	0.0001	0.094
RBC T_2_* (ms)	−0.025 × 10^−1^	−0.0044	−0.0006	0.009
Pulmonary blood volume (a.u.)	0.026	−0.030	0.083	0.350
Pulmonary blood volume spatial heterogeneity (a.u.)	0.030	−0.027	0.088	0.285
Pulmonary blood flow (a.u.)	0.272	0.015	0.53	0.039
Pulmonary blood flow spatial heterogeneity (a.u.)	0.241	0.010	0.493	0.032
Mean transit time (s)	−0.021	−0.038	−0.005	0.015
Mean transit time spatial heterogeneity(s)	−0.007	−0.010	−0.004	< 0.001

*Note:* Regression models are adjusted for age and sex unless already adjusted via a *z*‐score. Ventilation metrics are also adjusted for smoking status.

Abbreviations: FEV_1_: forced expiry volume in 1 s; FVC: forced vital capacity; KCO carbon monoxide transfer coefficient; LMD: mean diffusive length scale; M: membrane; PFT: pulmonary function tests; RBC: red blood cell; SD: standard deviation; TLCO: transfer factor for carbon monoxide; VDP: ventilation defect percentage.

Carbon monoxide gas transfer (TL_CO_ *z*‐score) was abnormal in 10/13 participants at 6 months, 13/18 participants at 1 year, and 8/10 participants at 2 years. TL_CO_ and K_CO_ did not significantly change with time indicating persistent gas transfer abnormalities for the majority of subjects.

### 3.4 MRI

**FIGURE 3:**
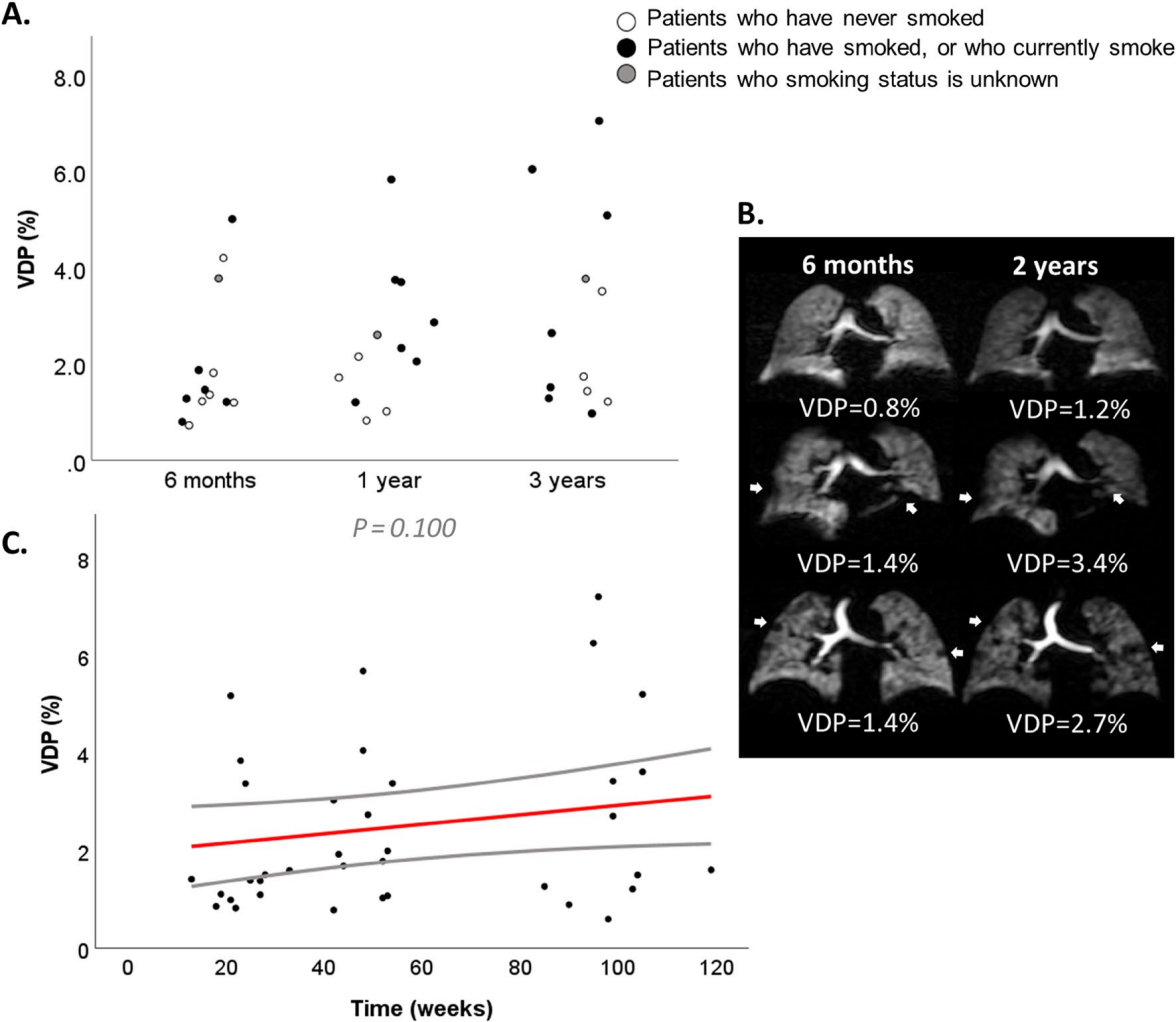
Summary of ventilation data: (A) a jitter plot of VDP at each visit, with participant color coded by their smoking status, (B) single central coronal slices from the lung ventilation images in three participant at 6 months and 2 years with whole‐lung VDP shown under each image and areas of ventilation defect indicated with white arrows, (C) a scatter plot of VDP with time with solid lines indicating the linear trend and confidence intervals.

^129^Xe lung ventilation imaging showed small peripheral ventilation defects and areas of low or heterogeneous ventilation (Figure 3). Median VDP was 1.4 (1.4)% at 6 months, 2.0 (2.1)% at 1 year, and 2.2 (3.6)% at 2 years. There was no overall change in lung ventilation metrics (VDP *p* = 0.100, low ventilation percentage *p* = 0.057, or ventilation heterogeneity index *p* = 0.194) with time (Table 3).

**FIGURE 4:**
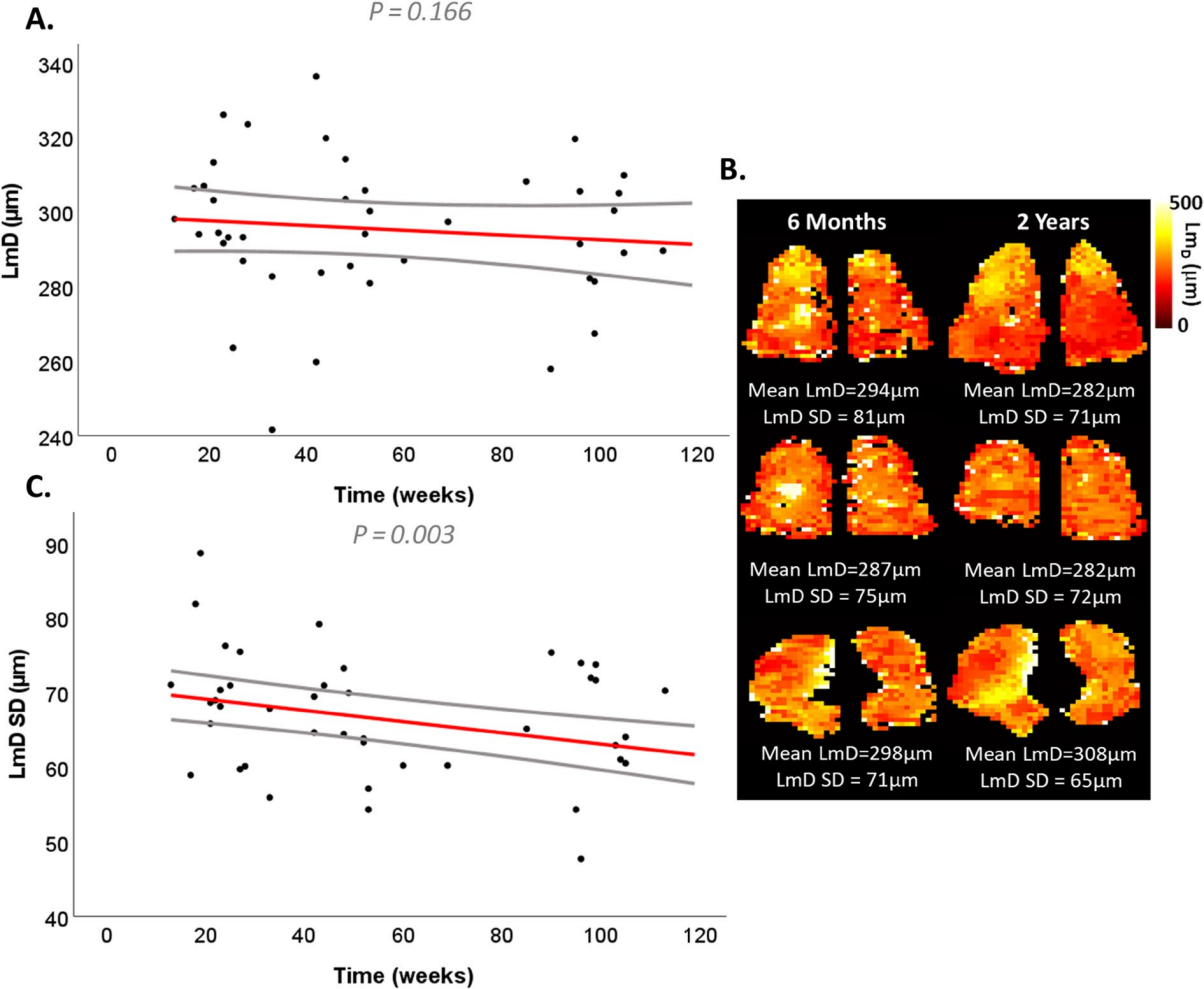
Summary of diffusion data: (A) a scatter plot of mean linear intercept, a measure of acinar airway dimensions (Lm_D_) with time, (B) a single representative coronal lung slice from Lm_D_ maps for three participant with diffusion‐weighted MRI acquired at both 6 months and 2 year time points, with mean and standard deviation of Lm_D_ from the whole lung shown under each representative slice, (C) A scatter plot of the standard deviation of Lm_D_ with time. Solid lines in (A) and (C) indicate the linear trend and confidence intervals predicted by the mixed‐effect linear regression analysis.

Diffusion weighted ^129^Xe MRI imaging showed that Lm_D_ did not change significantly with time (*p* = 0.166); however, the heterogeneity of acinar airway dimensions (Lm_D_ SD) decreased with time (Table 3, Figure 4).

**FIGURE 5:**
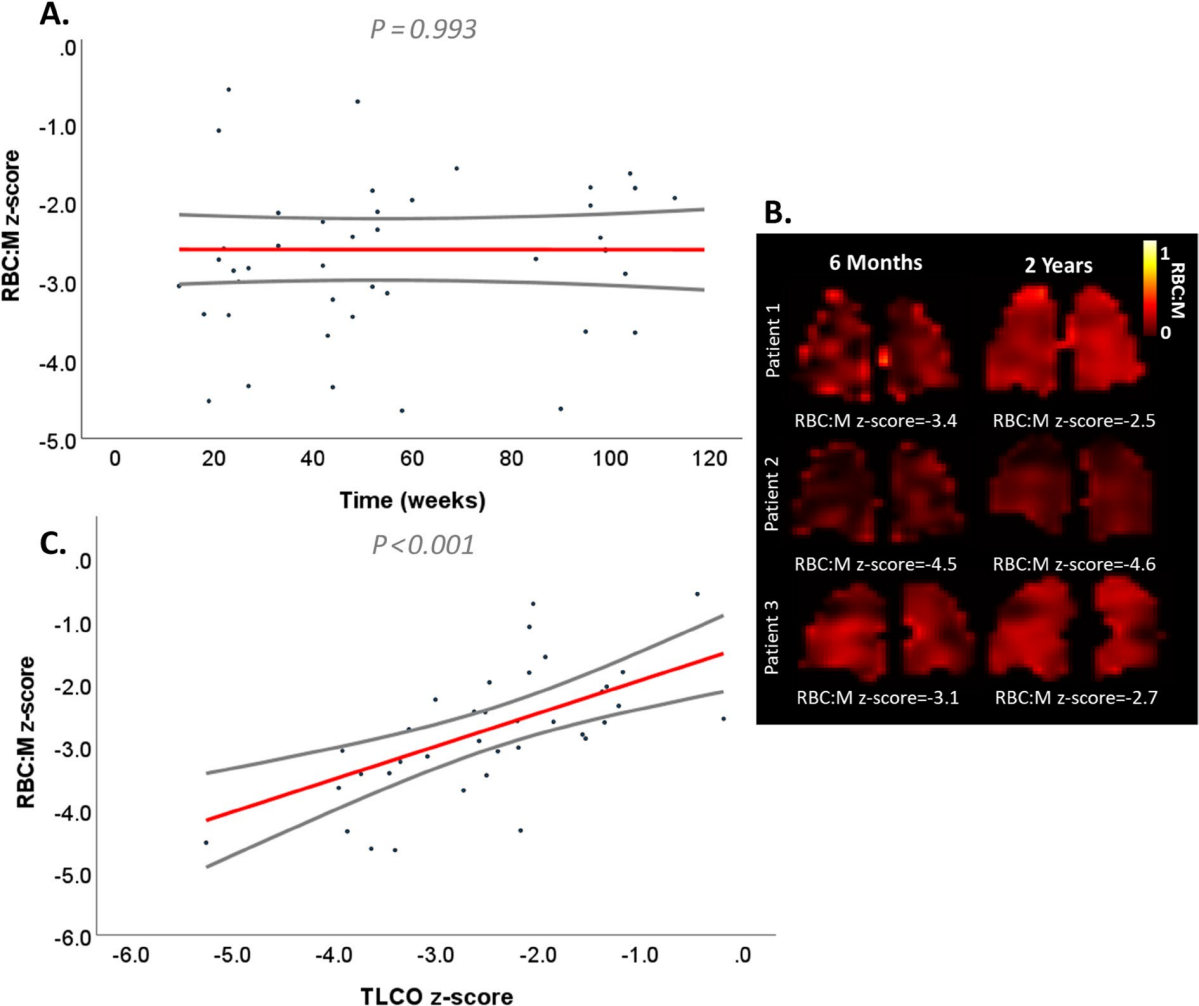
Summary of xenon gas transfer (RBC:M *z*‐score) data: (A) A scatter plot of RBC:M *z*‐score with time. (B) A single coronal slice from the RBC:M maps of three participant at 6 months and 2 years visits, two showing persistent upper lobe RBC:M defects and one showing improvement at 2 years, (C) a scatter plot of RBC:M *z*‐score and TL_CO_ *z*‐score. Solid lines in (A) and (C) indicate the linear trend and confidence intervals predicted by the mixed‐effect linear regression analysis.

Dissolved phase ^129^Xe xenon imaging showed RBC:M was abnormally low (median *z*‐score < −1.64) at all three visits (Table 2, Figure 5). In Figure 5, Participants 2 and 3 showed persistent RBC:M defects in the upper right lung. There was no significant change in RBC:M *z*‐score with time (*p* = 0.993). However, the T_2_* of the ^129^Xe in the RBCs (RBC T_2_*) significantly decreased with time (*p* = 0.009, Table 3).

M:gas *z*‐score was within the healthy range at all three visits; however, three participants had low M:gas (*z*‐score < −1.64) at one or more visits, and six participants had high M:gas at one or more visits (> 1.64). M:gas *z*‐score did not change significantly with time (*p* = 0.490, Table 3).

Box plots of MRI metrics acquired in participants hospitalized due to COVID‐19 with RLAs and previously published MRI metrics acquired in participants hospitalized due to COVID‐19 with normal structural lung imaging at 3 months after hospitalization [33] are shown in Figure S3.

**FIGURE 6:**
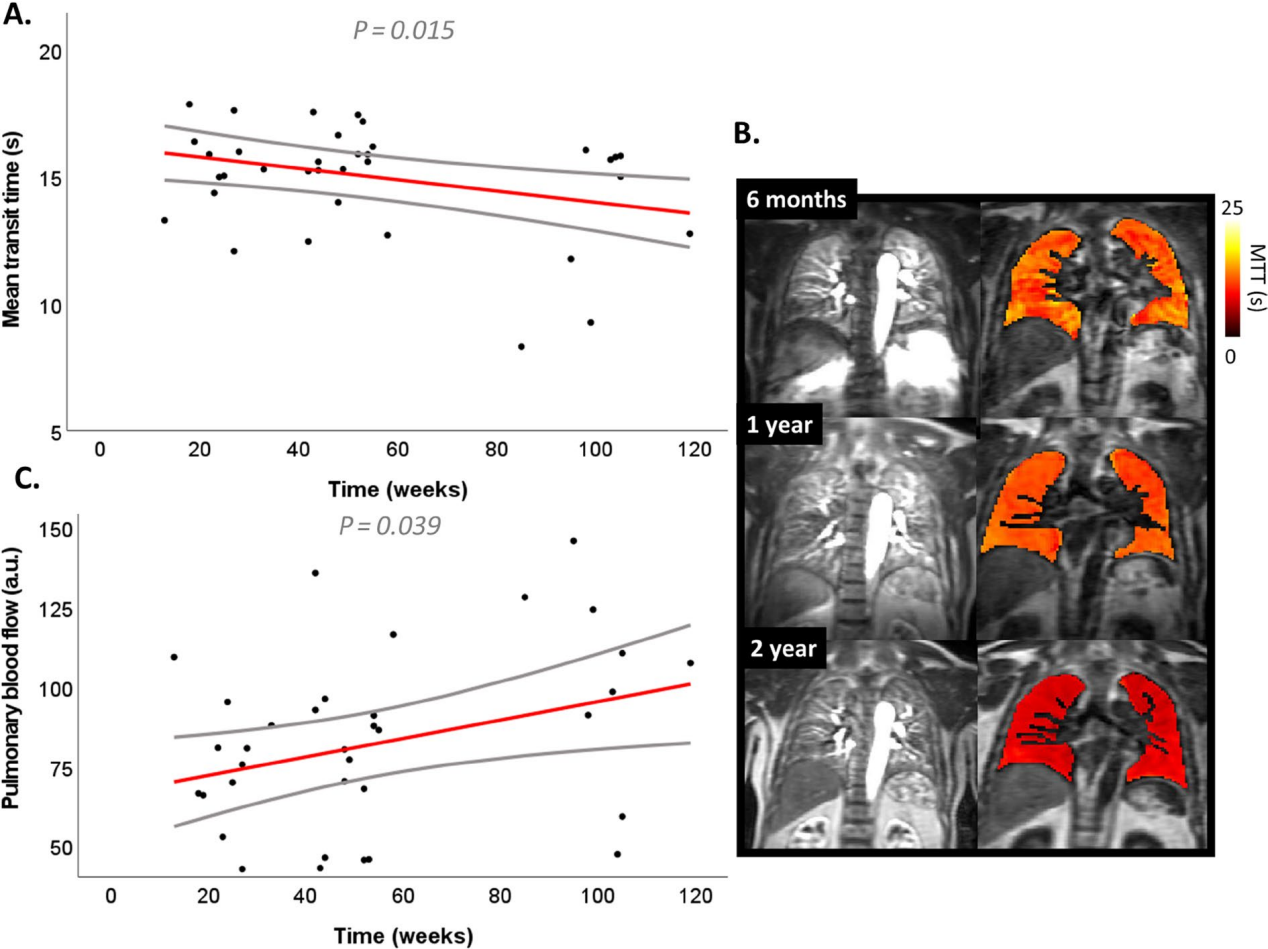
A summary of lung perfusion data: (A, C) scatterplots of pulmonary blood volume and mean transit time with time, with solid lines indicating the linear trend and confidence intervals predicted by the mixed‐effect linear regression analysis. (B) Example coronal slices from the peak perfusion maps (left, maximum signal intensity across all time points) and the maps of mean transit time (right, overlaid over the corresponding perfusion image) in a single patient at 6 months, 1 year, and 2 years.

Mean transit time significantly decreased with time and pulmonary blood flow significantly increased with time (Table 3 and Figure 6), indicating improvements in lung perfusion with time. There were no significant changes in pulmonary blood volume over time.

### 3.5 Associations Between MRI, Pulmonary Function, and Acute Disease Severity

As anticipated, xenon gas transfer between the membrane and RBCs (RBC:M) was significantly associated with TL_CO_ *z*‐score (see Figure 5) and K_CO_ *z*‐score.

Reduced FVC *z*‐score was associated with increased xenon uptake by the alveolar membrane (M:gas *z*‐score) and reduced TL_CO_ *z*‐score, suggesting that participants with lung restriction had membrane thickening and reduced gas transfer. However, FVC *z*‐score was not significantly associated with higher VDP.

Full details of significant associations of RBC:M *z*‐score, FVC *z*‐score, Lm_D_, and M:gas *z*‐score are shown in Table 4.

**TABLE 4:** Repeated measures mixed‐effect linear regression analysis of RBC:M *z*‐score, FVC *z*‐score, mean transit time and M:gas *z*‐score with the following metrics: RBC:M z‐score, M:gas *z*‐score, Lm_D_, VDP, pulmonary blood volume, pulmonary blood flow, mean transit time, FEV_1_ *z*‐score, FVC *z*‐score, TL_CO_ *z*‐score, K_CO_ *z*‐score.

Variable	Estimate (lower CI, upper CI)	*p*
Associations with RBC:M *z*‐score		
TL_CO_ *z*‐score	0.52 (0.28, 0.77)	< 0.001
K_CO_ *z*‐score	0.64 (0.30, 0.98)	< 0.001
Associations with FVC *z*‐score		
M:gas z‐score	−0.30 (−0.59, −0.02)	0.036
TL_CO_ *z*‐score	0.52 (0.27,0.78)	< 0.001
FEV_1_ *z*‐score	0.89 (0.71,1.07)	< 0.001
Associations with mean transit time		
Pulmonary blood flow (a.u.)	−0.050 (−0.068,‐0.031)	< 0.001
Associations with M:gas *z*‐score		
Lm_D_ (μm)	−0.021 (−0.040,‐0.002)	0.030
Mean transit time (s)	−0.11 (−0.21,‐0.03)	0.01
FEV_1_ *z*‐score	−0.46 (−0.82,‐0.09)	0.02
FVC *z*‐score	−0.45 (−0.82,‐0.08)	0.02

*Note:* Only significant results are shown.

Abbreviations: FEV_1_: forced expiry volume in 1 s; FVC: forced vital capacity; KCO carbon monoxide transfer coefficient; LMD: mean diffusive length scale; M: membrane; RBC: red blood cell; SD: standard deviation; TLCO: transfer factor for carbon monoxide; VDP: ventilation defect percentage.

At 6 months, participants with a longer stay in hospital had significantly lower FEV_1_ (*r* = −0.556), FVC (*r* = −0.699), TL_CO_ *z*‐score (*r* = −0.646), and Lm_D_ SD (*r* = 0.660) at 6 months. There were no other significant correlations between acute hospitalization admission length and MRI metrics or CT scores at 6 months.

### 3.6 Regional Variations in Lung Function MRI

Figure S4 shows example images and maps from four participants with different regional patterns of lung function changes despite abnormal gas transfer in all participants and demonstrates the heterogeneity in regional lung function patterns across the participants.

## 4 Discussion

This study conducted a comprehensive, longitudinal assessment of lung function using ^129^Xe and ^1^H MRI methods in patients with RLAs following hospitalization due to COVID‐19. The abnormalities identified in pulmonary gas transfer and perfusion did not progress between 6 months and 2 years after hospital discharge, suggesting that these participants are not at risk of progressive interstitial lung disease. While lung perfusion significantly improved in this period, gas transfer did not, and xenon gas transfer was not associated with pulmonary perfusion metrics, which suggests persistent underlying alveolar‐capillary membrane dysfunction.

Participants in this study were older and predominantly male, which is consistent with the characteristics of patients most at risk of hospitalization and more severe acute COVID‐19 [1]. The participants in this study had reduced TL_CO_ and RBC:M, slightly increased M:gas, and reduced FEV_1_ and FVC, a pattern consistent with a cluster of long COVID patients previously identified with increased ^129^Xe membrane uptake, ground glass opacities on CT, restrictive PFT pattern and low RBC:M [34].

Dissolved phase xenon imaging enabled gas transfer between the airspace, membrane, and pulmonary capillaries (RBC) to be measured separately, providing additional insight into the patterns of gas transfer in patients with RLAs following COVID‐19. While abnormalities in xenon gas transfer to the capillaries (RBC:M) were consistent, 6/24 patients (25%) also had high M:gas during at least one visit, consistent with a pattern of “diffusion block” due to membrane thickening [35]. This is broadly consistent with previous findings of RBC:M in patients with idiopathic pulmonary fibrosis (IPF) [35, 36] who have been reported to demonstrate a variety of different patterns of gas transfer, including patients with membrane thickening alongside impaired RBC transfer, but also patients with low or normal membrane transfer but reduced RBC transfer [35].

RBC:M did not change with time which is consistent with previous longitudinal findings in patients after COVID‐19 in a mixed ambulatory and hospitalized cohort [17], with median RBC:M and RBC:gas lower in this cohort of participants than in patients previously hospitalized due to COVID‐19 without RLAs [33]. Previous work has shown correlation between DL_CO_ and perfusion abnormalities on dual‐energy CT in patients with long COVID 6 months after acute infection [37]. However, this work found mean transit time and pulmonary blood flow improved across the same time period. Improvement of pulmonary blood flow without improvement in gas transfer efficiency may occur if blood flow improves within an area of lung that is either unventilated (resulting in worsened VQ matching) or diffusion limited (due to thickened or impaired alveolar capillary membrane). In this study, both ventilation abnormalities and membrane thickening were identified in subsets of patients, and for those patients this is a plausible explanation for these findings. However, methodological differences may also play a part in explaining this disparity, as perfusion imaging visualizes blood flow across the entire pulmonary vascular bed, whereas xenon only dissolves in RBCs within the pulmonary capillaries.

Participants in this study had minor ventilation abnormalities that did not change with time. Previous findings on ventilation abnormalities after COVID‐19 are varied, with some findings showing a higher burden of ventilation abnormalities after COVID‐19 [12, 16, 38] and others reporting a low burden [33, 34, 39]. Reasons for this may include differences in pre‐existing ventilation abnormalities and respiratory comorbidities. FEV_1_ *z*‐score and FVC *z*‐score increased between 6 months and 2 years; however, VDP was not significantly associated with FVC *z*‐score and did not increase over the same period. Therefore, the improvements seen in FEV_1_ and FVC *z*‐scores are not due to an increase in ventilated lung, but instead either represent improvements in lung compliance or improvements in respiratory muscle strength.

Participants in this study did not have increased Lm_D_ compared to data on age and sex trends in a healthy cohort [40] and had smaller acinar airway dimensions (Lm_D_) than patients with IPF of a similar age to the patients in this study [41]. Patients with inflammatory interstitial lung disease have been shown to have similar RBC:M but decreased Lm_D_ when compared to patients with fibrotic interstitial lung disease [42]. In addition, the participants in this study have an overall improvement in FVC *z*‐score, suggesting resolving rather than irreversible lung damage. Together, this suggests that the participants in this study represent an inflammatory rather than fibrotic profile, which is also consistent with the higher prevalence of ground glass opacities than fibrosis seen on CT. Similarly, improvements in pulmonary perfusion metrics suggest microvascular recovery and align more closely with an inflammatory rather than a fibrotic phenotype.

### 4.1 Limitations

This study faced challenges in participant recruitment, retention, and data missingness, resulting in a small sample size, which is a key limitation of this work. The primary reason for the low recruitment was the progressive reduction in hospital admission rates for severe COVID‐19 as the pandemic evolved, yielding fewer patients who met the inclusion criteria.

We therefore performed a linear mixed model analysis, adjusted for participant age and sex where appropriate. While it was assumed that data missingness was random, it is possible that participants who did not experience improvement over the 18‐month time frame were more motivated to continue with the study, leading to an underestimation of potential improvement. Therefore, the statistical results should be interpreted cautiously as this may weaken the statistical power of the study. Despite these limitations, the findings clearly indicate that participants did not deteriorate over the study period. Further, only limited symptom data were recorded, and therefore a more thorough exploration of the link between lung function and symptom recovery could not be performed. Finally, a further limitation is that CT images were read individually at each site.

## 5 Conclusion

In patients hospitalized due to COVID‐19 with RLAs, improvements with time were seen in lung volume and perfusion; however, xenon gas transfer and TL_CO_ remained abnormally low with no change observed between 6 months and 2 years. This persistent gas transfer impairment, despite pulmonary perfusion recovery, suggests that underlying alveolar‐capillary membrane dysfunction may not fully resolve, indicating potential long‐term consequences for gas exchange efficiency and highlights the need for treatments that target this deficit.

## Funding

This independent research was carried out at the National Institute for Health and Care Research (NIHR) Sheffield Biomedical Research Centre (BRC), NIHR Nottingham Biomedical Research Centre, and NIHR Oxford Biomedical Research Centre. This study was supported by NIHR UCLH Biomedical Research Centre.

The UK Interstitial Lung Disease Long‐COVID19 study (UKILD‐Long COVID): understanding the burden of Interstitial Lung Disease in Long COVID (Medical Research Council, MR/W006111/1). Expansion of state‐of‐the‐art MR imaging infrastructure for pulmonary disease stratification: POLARIS (Medical Research Council, MR/M008894/1), INSIGNEO Sheffield, J.J. was funded by the Wellcome Trust. J.J. and by the NIHR UCLH Biomedical Research Centre. IPH is an NIHR Senior Investigator. The views expressed are those of the author(s) and not necessarily those of the MRC, Sheffield, Oxford, Nottingham, or UCLH BRC or the Department of Health and Social Care.

## Supporting information


**Table S1:** Typical imaging parameters used for MRI acquisition.
**Table S2:** Visualization of patient recruitment and missing data.
**Figure S1:** Histograms of CT scores for (A) fibrotic‐like changes, (B) reticulation, (C) ground glass opacities (GGO), and (D) whole lung CT score, where a maximum score is 25.
**Figure S2**: Scatterplots of CT scores and 6‐month MRI and PFT data. Specifically: a) ground glass opacity CT score and KCO z‐score, b) reticulation CT score and FVC z‐score, c) fibrosis CT score and TLCO z‐score and D) total CT score and RBC T_2_*.
**Figure S3:** Showing Lm_D_ (A), VDP (B), RBC:M *z*‐score (C), M:gas *z*‐score (D), and RBC:gas *z*‐score (E) at 6‐month and 1‐year time points in patients hospitalized due to COVID‐19 with RLAs, alongside previously published data acquired in patients hospitalized due to COVID‐19 with normal lung structural imaging at 3 months after hospitalization (patients without RLAs) (8).
**Figure S4:** Example ultra‐short echo time (UTE) and ventilation images, and Lm_D_, RBC:M, M:gas, and MTT maps for four patients with different patterns of regional alignment between functional metrics.
