# Supplementary material for: Longitudinal 1H and 129Xe Lung MRI in Patients With Post‐COVID Residual Lung Abnormalities
Source: J Magn Reson Imaging. 2026 May 28;64(3):669–82. doi: 10.1002/jmri.70368 (PMC13466584; doi:10.1002/jmri.70368)
Supplement: Supplementary file 1 [file JMRI-64-669-s001.docx]

Online Data Supplement

**Title:** Longitudinal assessment of ^1^H and ^129^Xe lung MRI in patients with post-COVID residual lung abnormalities.

**Methods:**

Pulmonary function tests

Forced expiratory volume in 1 second (FEV_1_), forced vital capacity (FVC), FEV_1_/FVC, the transfer factor for carbon monoxide (TL_CO_) and the carbon monoxide transfer coefficient (K_CO_) were measured and presented as z-scores(1, 2)

**Results**

Details of missing data are shown in Table E2. Patient’s whose visits were at Site 3 or Site 4 did not have ventilation MRI or perfusion MRI and nearest PFT to the research visit was used where possible. Patients whose visits were at Site 1 had a research PFT, acquired on the same day as MRI where possible.

Table S1**:** Typical imaging parameters used for MRI acquisition

|  | **^129^Xe ventilation** | **^129^Xe DW-MRI** | **Dissolved phase ^129^Xe** | **^1^H dynamic contrast enhanced perfusion** |
| --- | --- | --- | --- | --- |
| Sequence | 3D steady state free precession sequence (4) | 3D spoiled gradient echo multiple b-value with elliptical-centric phase encoding (5) | 4-echo flyback 3D radial technique (6) | 3D spoiled gradient echo |
| Acquired matrix | 78x80 | 64 × 52 | 20x20x20 | 200x80 |
| Field of view (cm^2^) | 48x38 | 48x38 | 40x40 | 48x48 |
| Slice thickness (mm) | 10 | 20 | 0.55 | 10 |
| TE (ms) | 1.4 | 14.0 | 0.57/1.27/1.97/2.67 | 0.72 |
| TR (ms) | 4.0 | 17.4 | 15 | 1.99 |
| FA (°) | 10 | 3 | 22/0.22 | 20° |
| Temporal acquisitions | - | - | - | 48 |
| Temporal resolution (s) | - | - | - | ~0.5 |
| SENSE factor | - | - | - | 2 |
| Xenon dose (mL) | 500 | 550 | 1000 | - |
| Respiratory state | Functional residual capacity + maximum 1L bag of gas (^129^Xe and N_2_ mixture), titrated according to height(7) | Functional residual capacity + 1L bag of gas (^129^Xe and N_2_ mixture), titrated according to height(7) | Functional residual capacity + 1L bag of gas (^129^Xe), titrated according to height(7) | Expiration breath hold followed by shallow breathing |
| Additional parameters |  | b = 0, 12, 20, 30 s/cm^2^  diffusion time (Δ) = 8.5 ms | ΔTE = 0.7 | ^[1]^ |

Table S2: Visualisation of patient recruitment and missing data. Red boxes indicate the patient attended the visit, but the data was not acquired. Orange boxes indicate the patient attended the visit, data was acquired, but did not pass quality control. Green boxes indicate data was acquired and used during analysis. 1: Oxford. 2: Sheffield. 3: Nottingham

|  |  | Visit 1 | | | | | Visit 2 | | | | | Visit 3 | | | | |
| --- | --- | --- | --- | --- | --- | --- | --- | --- | --- | --- | --- | --- | --- | --- | --- | --- |
|  | Site | Vent | Dp | Diff | Perf | PFT | Vent | Dp | Diff | Perf | PFT | Vent | Dp | Diff | Perf | PFT |
| 01 | 1 |  |  |  |  |  |  |  |  |  |  |  |  |  |  |  |
| 02 | 1 |  |  |  |  |  |  |  |  |  |  |  |  |  |  |  |
| 03 | 1 |  |  |  |  |  |  |  |  |  |  |  |  |  |  |  |
| 04 | 2 |  |  |  |  |  |  |  |  |  |  |  |  |  |  |  |
| 05 | 2 |  |  |  |  |  |  |  |  |  |  |  |  |  |  |  |
| 06 | 2 |  |  |  |  |  |  |  |  |  |  |  |  |  |  |  |
| 07 | 2 |  |  |  |  |  |  |  |  |  |  |  |  |  |  |  |
| 08 | 2 |  |  |  |  |  |  |  |  |  |  |  |  |  |  |  |
| 09 | 2 |  |  |  |  |  |  |  |  |  |  |  |  |  |  |  |
| 10 | 2 |  |  |  |  |  |  |  |  |  |  |  |  |  |  |  |
| 11 | 2 |  |  |  |  |  |  |  |  |  |  |  |  |  |  |  |
| 12 | 2 |  |  |  |  |  |  |  |  |  |  |  |  |  |  |  |
| 13 | 2 |  |  |  |  |  |  |  |  |  |  |  |  |  |  |  |
| 14 | 2 |  |  |  |  |  |  |  |  |  |  |  |  |  |  |  |
| 15 | 2 |  |  |  |  |  |  |  |  |  |  |  |  |  |  |  |
| 16 | 2 |  |  |  |  |  |  |  |  |  |  |  |  |  |  |  |
| 17 | 2 |  |  |  |  |  |  |  |  |  |  |  |  |  |  |  |
| 18 | 2 |  |  |  |  |  |  |  |  |  |  |  |  |  |  |  |
| 19 | 2 |  |  |  |  |  |  |  |  |  |  |  |  |  |  |  |
| 20 | 2 |  |  |  |  |  |  |  |  |  |  |  |  |  |  |  |
| 21 | 2 |  |  |  |  |  |  |  |  |  |  |  |  |  |  |  |
| 22 | 3 |  |  |  |  |  |  |  |  |  |  |  |  |  |  |  |
| 23 | 3 |  |  |  |  |  |  |  |  |  |  |  |  |  |  |  |
| 24 | 3 |  |  |  |  |  |  |  |  |  |  |  |  |  |  | ^[2]^ |

Figure S1: Histograms of CT scores for A) fibrotic-like changes B) reticulation, C) ground glass opacities (GGO)and D) whole lung CT score, where a maximum score is 25.

Figure S2: Scatterplots of CT scores and 6 month MRI and PFT data. Specifically: a) ground glass opacity CT score and K_CO_ z-score, b) reticulation CT score and FVC z-score, c) fibrosis CT score and TL_CO_ z-score and d) total CT score and RBC T_2_^*^.

Figure S3: Showing Lm_D_ (A), VDP (B), RBC:M z-score (C), M:gas z-score (D), and RBC:gas z-score (E) at 6-months and 1-year time points in patients hospitalised due to COVID-19 with RLAs, alongside previously published data acquired in patients hospitalised due to COVID-19 with normal lung structural imaging at 3 months after hospitalisation (patients without RLAs) (8). Data shown are from 9 patients without RLAs, 7 of which attended 6 month MRI and 8 of which attended 1 year MRI with 6 patients present at both visits. RBC:M, RBC:gas and M:gas are expressed as z-scores.

Figure S4: Example ultra-short echo time (UTE) and ventilation images, and LmD, RBC:M, M:gas and MTT maps for 4 patients with different patterns of regional alignment between functional metrics. Patient A is 6 months post discharge and has lower lung structural abnormalities [A1] which align with lung ventilation heterogeneity[A2], increased lung microstructure (LmD) [A3], decreased xenon gas transfer (RBC:M)[A4] and prolonged transit times (MTT)[A6] in the lower lung. Xenon membrane uptake (M:gas) is not increased in the lower lung [A5]. Patient B is 2 years post discharge and has increased lung microstructure (LmD)[B3] and increased uptake of xenon in the membrane (M:gas) [B5] in the left lung corresponding to an area of structural lung abnormality[B1], with minimal corresponding change in lung perfusion (MTT)[B6]. Patient C is 6 months post discharge and shows largely homogeneous lung microstructure (LmD) [C5] and perfusion (MTT) [C6] despite reduced xenon gas transfer (RBC:M z-score=-2.56) [C4] and some heterogeneity in xenon membrane uptake (M:gas)[C5]. Patient D is 6 months post discharge and has increased lung microstructure (LmD) [D3] in the upper right lung, peripherally increased uptake of xenon in the membrane (M:gas) [D5] in the left lung, and homogeneously prolonged transit times (MTT) [D6] with little visual concordance between abnormal regions seen in each image or map.

**OLS References:**

1. Quanjer PH, Stanojevic S, Cole TJ, Baur X, Hall GL, Culver BH, Enright PL, Hankinson JL, Ip MS, Zheng J, Stocks J, Initiative ERSGLF. Multi-ethnic reference values for spirometry for the 3-95-yr age range: the global lung function 2012 equations. *Eur Respir J* 2012; 40: 1324-1343.

2. Stanojevic S, Graham BL, Cooper BG, Thompson BR, Carter KW, Francis RW, Hall GL, Global Lung Function Initiative Twg, Global Lung Function Initiative T. Official ERS technical standards: Global Lung Function Initiative reference values for the carbon monoxide transfer factor for Caucasians. *Eur Respir J* 2017; 50.

3. Knight SR, Ho A, Pius R, Buchan I, Carson G, Drake TM, Dunning J, Fairfield CJ, Gamble C, Green CA, Gupta R, Halpin S, Hardwick HE, Holden KA, Horby PW, Jackson C, McLean KA, Merson L, Nguyen-Van-Tam JS, Norman L, Noursadeghi M, Olliaro PL, Pritchard MG, Russell CD, Shaw CA, Sheikh A, Solomon T, Sudlow C, Swann OV, Turtle LC, Openshaw PJ, Baillie JK, Semple MG, Docherty AB, Harrison EM, investigators IC. Risk stratification of patients admitted to hospital with covid-19 using the ISARIC WHO Clinical Characterisation Protocol: development and validation of the 4C Mortality Score. *BMJ* 2020; 370: m3339.

4. Stewart NJ, Norquay G, Griffiths PD, Wild JM. Feasibility of human lung ventilation imaging using highly polarized naturally abundant xenon and optimized three-dimensional steady-state free precession. *Magn Reson Med* 2015; 74: 346-352.

5. Chan HF, Stewart NJ, Norquay G, Collier GJ, Wild JM. 3D diffusion-weighted (129) Xe MRI for whole lung morphometry. *Magn Reson Med* 2018; 79: 2986-2995.

6. Collier GJ, Eaden JA, Hughes PJC, Bianchi SM, Stewart NJ, Weatherley ND, Norquay G, Schulte RF, Wild JM. Dissolved (129) Xe lung MRI with four-echo 3D radial spectroscopic imaging: Quantification of regional gas transfer in idiopathic pulmonary fibrosis. *Magn Reson Med* 2021; 85: 2622-2633.

7. Smith LJ, Collier GJ, Marshall H, Hughes PJC, Biancardi AM, Wildman M, Aldag I, West N, Horsley A, Wild JM. Patterns of regional lung physiology in cystic fibrosis using ventilation magnetic resonance imaging and multiple-breath washout. *Eur Respir J* 2018; 52.

8. Saunders LC, Collier GJ, Chan HF, Hughes PJC, Smith LJ, Watson JGR, Meiring JE, Gabriel Z, Newman T, Plowright M, Wade P, Eaden JA, Thomas S, Strickland S, Gustafsson L, Bray JB, Marshall H, Capener DA, Armstrong L, Rodgers J, Brook M, Biancardi AM, Rao MR, Norquay G, Rodgers O, Munro R, Ball JE, Stewart NJ, Lawrie A, Jenkins RG, Grist JT, Gleeson F, Schulte RF, Johnson KM, Wilson FJ, Cahn A, Swift AJ, Rajaram S, Mills GH, Watson L, Collini PJ, Lawson R, Thompson AAR, Wild JM. Longitudinal Lung Function Assessment of Patients Hospitalized With COVID-19 Using (1)H and (129)Xe Lung MRI. *Chest* 2023; 164: 700-716.

1. DW-MRI: Diffusion-weighted 129Xe MRI; TE: echo time; TR: repetition time; FA: flip angle.
2. Vent: ventilation imaging; Dp: dissolved phase imaging; Perf: perfusion imaging; PFT: pulmonary function tests
